# Supplementary material for: Surface markers on supermeres outperform extracellular vesicles in colorectal cancer diagnosis
Source: Sci Rep. 2026 Jan 22;16:5989. doi: 10.1038/s41598-026-36626-8 (PMC12902025; doi:10.1038/s41598-026-36626-8)
Supplement: Supplementary file 1 — Supplementary Material 1 [file 41598_2026_36626_MOESM1_ESM.pdf]

Supplementary Material for

Surface Markers on Supermeres Outperform Extracellular Vesicles in Colorectal Cancer Diagnosis

Sonu Kumar[1], John Alex Sinclair[1], Tiger Shi[1], Gauen Kim[1], Runyao Zhu[1], Grace Gasper[1], Yichun Wang[1], James N. Higginbotham[2], Qin Zhang[2], Dennis K. Jeppesen[2], Oleg Tutanov[2], Maxwell Hamilton[4], Jeffrey L. Franklin[2,3], Al Charest[5,6], Robert J. Coffey[2,3], Satyajyoti Senapati[1]*, Hsueh-Chia Chang[1]*

[1]Department of Chemical and Biomolecular Engineering, University of Notre Dame, Notre Dame, IN 46556, USA
[2]Department of Medicine, Vanderbilt University Medical Center, Nashville, TN 37232, USA
[3]Department of Cell and Developmental Biology, Vanderbilt University School of Medicine, Nashville, TN 37232, USA
[4]Program in Cancer Biology, Vanderbilt University School of Medicine, Nashville, TN, 37232, USA
[5]Department of Medicine, Beth Israel Deaconess Medical Center, Harvard Medical School, Boston, MA, USA
[6]Cancer Research Institute, Beth Israel Deaconess Medical Center, Boston, MA, USA

Corresponding Authors: hchang@nd.edu (H. -C. Chang) & ssenapat@nd.edu (S. Senapati)

Abstract: Extracellular nanocarriers, such as extracellular vesicles (EVs), lipoproteins, supermeres, and exomeres are diverse lipid-protein-nucleic acid assemblies. Among them, supermeres hold significant diagnostic potential but are challenging to characterize due to limited surface biomarker information and labor-intensive isolation methods. This study introduces an isolation-free Ion Exchange Membrane Sensing method for the detection of supermeres within 30 minutes using 50 µL of sample, with a sensitivity of 10^6–10^7 supermeres/mL. Validation through ultracentrifugation (UC) and surface plasmon resonance (SPR) confirms the detection accuracy and specificity. Supermeres carry key proteins such as HSPA13, ENO2, and DDR1 analogous to tetraspanin in EV. Supermeres outperform small EVs (sEVs) and exomeres across multiple shared and unique surface proteins critical to colorectal cancer diagnosis, highlighting their superior clinical utility and potential as next-generation biomarkers in precision medicine.

Estimating Voltage-Shift Signal Dependence on IEM Sensor Membrane Size

It is difficult to fabricate IEM sensors of the same size, and hence the signal needs to be properly corrected for variations in the membrane size. In earlier reports (*44,45*), we and others have shown that, for an ideally selective membrane and a symmetric electrolyte with equal diffusivity and valency, the electromigration flux of the counterions is equal to its diffusive flux and hence the total ion flux is twice the diffusive flux. At the limiting current condition for a small membrane (or its nanoslot model), an ion-depleted region exists on the surface of the membrane with a dimension close to the size (radius) R of the membrane (*46*). The finite size of the depleted region is due to radial focusing of the diffusive flux to a point-like sink that is the membrane sensor. It is related to the fundamental solution 1/r of the diffusion equation in spherical coordinates. This known depletion or diffusion length allows us to estimate the limiting ion flux density as $2Dc_0/R$ and the Levich limiting current density i_{lim} as $\frac{2zFDc_0}{R}$. The limiting current is very easy to measure for each sensor, as it corresponds to a distinct voltage-independent current plateau of the I-V curve (see Figure 2b in the text). The voltage shift is measured at the onset of the over-limiting current (*47*) from the limiting current, where this relationship still holds (*48*). Since the over-limiting current, I-V curve is linear, the inverse scaling with respect to the membrane size R also holds for the voltage shift. In Supplementary Figure 1, we verify this R^{-1} relationship between the voltage shift observed and the size R of the membrane given as $\sim i_{lim}/2DzFc_0$ where i_{lim} is the limiting current density, D is the diffusivity of the ions ($1.5 \times 10^{-9}\ m^2/s$), z is the valency of the ions ($z = 1$), F is the Faraday constant and c_0 is the ionic concentration of the bulk (10 mM) during IV measurement.

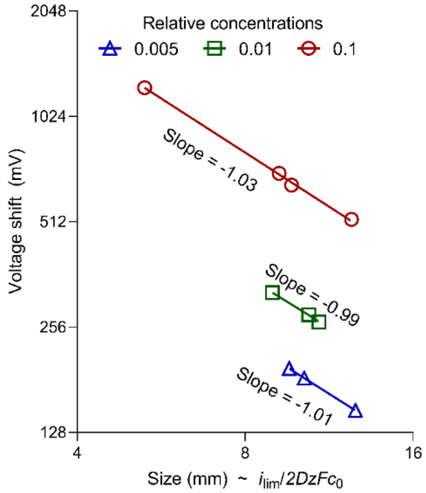

Figure S1. Size-dependence on the theoretical IEMS size of the voltage shift observed with respect to different stock concentrations of supermeres at different dilutions using anti-TGFBI antibody

The inverse linear relationship between the voltage shift and theoretical size of the IEMS sensor R suggests an opposing electric field generated by our analyte at a distance of about one membrane sensor distance, which is also the radius of our ion depletion zone. In this region, the ionic concentration reaches the lower asymptote of deionized water-like condition (10^{-7} M). Therefore, our signal is thus defined as representative of this charge given as (with initial state representing pre-incubation and final state representing incubation followed by wash), with V_1 and V_2 measured at the 2x limiting current for reference and to ensure we are in the over-limiting region. The parameter ϵ_0 represents the permittivity of vacuum and ϵ_r the relative permittivity of water (~80):

$$\frac{q_{initial}}{4\pi\epsilon_0\epsilon_r R} = V_1$$

$$\frac{q_{final}}{4\pi\epsilon_0\epsilon_r R} = V_2$$

These equations again verify that the voltage shift scales inversely with R, with a proportionality constant that corresponds to the charge of the captured analyte. Therefore, the membrane size R dependence of the charge signal S can be replaced by the total limiting current $I_{lim} = \pi R^2 i_{lim} = 2zFDc_0\pi R$:

$$S = q_{final} - q_{initial} = 4\pi\epsilon_0\epsilon_r R(V_2 - V_1) = \frac{2\epsilon_0\epsilon_r I_{lim}(V_2 - V_1)}{DzFc_0}$$

Here, $V_2 - V_1$ is essential for our voltage shift ΔV, so we can write it in final form as:

$$S = \frac{2\epsilon_0\epsilon_r I_{lim}\Delta V}{DzFc_0}$$

The value of I_{lim} is obtained from the I-V curve of every measurement.

Absence of apolipoprotein A1 in different nanocarrier fractions

We have already shown that different markers are enriched in different fractions. Here, we show that apoliproteins are significantly depleted in our isolated fractions. Figure S2 shows apolipoprotein A1 (ApoA1) measured using ELISA in pooled plasma, showing significant depletion.

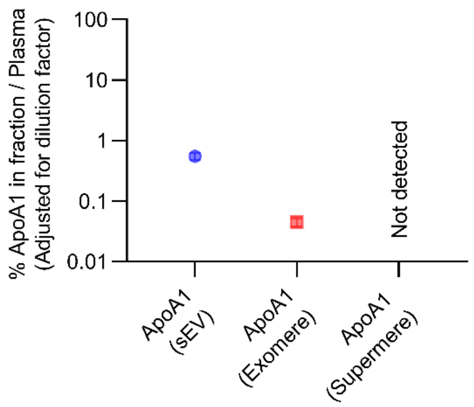

Figure S2. Relative concentration of ApoA1 in different nanocarrier fractions compared to plasma concentration.

References

44. V. G. Levich. Physicochemical Hydrodynamics. Prentice-Hall, Englewood Cliffs, NJ (1962).

45. H.-C. Chang, L. Y. Yeo. Electrokinetically driven microfluidics and nanofluidics. *Cambridge University Press (2010)*.

46. G. Yossifon, P. Mushenheim, Y. C. Chang, H.-C. Chang. Eliminating the limiting-current phenomenon by geometric field focusing into nanopores and nanoslots. *Physical Review E—Statistical, Nonlinear, and Soft Matter Physics*, **81**, 046301 (2010). doi: 10.1103/PhysRevE.81.046301; PMID: 19113713

47. G. Yossifon, H. -C. Chang. Selection of nonequilibrium overlimiting currents: universal depletion layer formation dynamics and vortex instability. *Physical Review Letters*, **101,** 254501 (2008). doi: 10.1103/PhysRevLett.101.254501; PMID: 19113713

48. S. Sensale, Z. Ramshani, S. Senapati, H.-C. Chang. Universal features of non-equilibrium ionic currents through perm-selective membranes: gating by charged nanoparticles/macromolecules for robust biosensing applications. *The Journal of Physical Chemistry B*, ***125*****,** 1906-1915 (2001). doi: 10.1021/acs.jpcb.0c09916; PMID: 33410691
